# The ecological impact of city lighting scenarios: exploring gap crossing thresholds for urban bats

**DOI:** 10.1111/gcb.12884

**Published:** 2015-03-06

**Authors:** James D. Hale, Alison J. Fairbrass, Thomas J. Matthews, Gemma Davies, Jon P. Sadler

**Affiliations:** ^1^ School of Geography, Earth and Environmental SciencesUniversity of BirminghamEdgbastonBirminghamB15 2TTUK; ^2^ Centre for Urban Sustainability and ResilienceUniversity College LondonChadwick BuildingGower StreetLondonWC1E 6BTUK; ^3^ School of Geography and the EnvironmentOxford University Centre for the EnvironmentUniversity of OxfordSouth Parks RoadOxfordOX1 3QYUK; ^4^ CE3C – Centre for Ecology, Evolution and Environmental Changes/Azorean Biodiversity Group and Universidade dos AçoresDepartamento de Ciências AgráriasPortugal; ^5^ Lancaster Environment CentreLancaster UniversityLancasterLA1 4QYUK

**Keywords:** connectivity, gap crossing, lighting, movement, *Pipistrellus pipistrellus*, scenarios, urban, urbanization

## Abstract

As the global population urbanizes, dramatic changes are expected in city lighting and the urban form, which may threaten the functioning of urban ecosystems and the services they deliver. However, little is known about the ecological impact of lighting in different urban contexts. Movement is an important ecological process that can be disrupted by artificial lighting. We explored the impact of lighting on gap crossing for *Pipistrellus pipistrellus*, a species of bat (Chiroptera) common within UK cities. We aimed to determine whether the probability of crossing gaps in tree cover varied with crossing distance and lighting level, through stratified field surveys. We then used the resulting data on barrier thresholds to model the landscape resistance due to lighting across an entire city and explored the potential impact of scenarios for future changes to street lighting. The level of illumination required to create a barrier effect reduced as crossing distance increased. For those gaps where crossing was recorded, bats selected the darker parts of gaps. Heavily built parts of the case study city were associated with large and brightly lit gaps, and spatial models indicate movement would be highly restricted in these areas. Under a scenario for brighter street lighting, the area of accessible land cover was further reduced in heavily built parts of the city. We believe that this is the first study to demonstrate how lighting may create resistance to species movement throughout an entire city. That connectivity in urban areas is being disrupted for a relatively common species raises questions about the impacts on less tolerant groups and the resilience of bat communities in urban centres. However, this mechanistic approach raises the possibility that some ecological function could be restored in these areas through the strategic dimming of lighting and narrowing of gaps.

## Introduction

Urban areas are now home to over half of the world's population (UN, 2010), are the drivers behind much of the global CO_2_ emissions and resource demands (Wackernagel *et al*., 2006; Hoornweg *et al*., 2011) and are highly modified environments (Grimm *et al*., 2008). They are therefore at the heart of debates about climate change, resource security, nature conservation, and human well‐being (Newman, 2006; Grimm *et al*., 2008; Hodson & Marvin, 2009; Glaeser, 2011). Given the diversity and complexity of change within urban areas (Dallimer *et al*., 2011), there is a need to explore how their sustainability performance might vary under alternative scenarios for their future structure and operation (Lombardi *et al*., 2012). In this study, we explore how the disruption of the nocturnal urban environment by different levels of artificial lighting can impact species movement – a key ecological process.

Growth, sprawl, compaction, and fragmentation of the built form varies within and between urban areas (Williams, 1999; Luck & Wu, 2002; Couch *et al*., 2005; Irwin & Bockstael, 2007; Adams *et al*., 2010; Seto *et al*., 2011), and changes in built extent, density, and land use may occur over relatively short time periods (Pauleit *et al*., 2005; Seto & Fragkias, 2005; Dallimer *et al*., 2011). In addition to shifts in urban form, changing technologies and social practices also radically alter urban environments (Gandy, 2004). One important example is outdoor artificial lighting, a pervasive yet diverse characteristic of cities that is changing in many regions (Bennie *et al*., 2014a; Kyba *et al*., 2014). Remotely sensed measures of light emissions from the earth's surface have been found to correlate with built land cover (Hale *et al*., 2013), population density (Sutton *et al*., 1997), electric power consumption (Elvidge *et al*., 1997), and per capita income (Ebener *et al*., 2005). Outdoor artificial lighting also varies considerably within cities depending on land cover and land use (Luginbuhl *et al*., 2009; Kuechly *et al*., 2012; Hale *et al*., 2013; Levin *et al*., 2014). Intensification and expansion of lighting is evident at both local and global scales (Hölker *et al*., 2010a; Bennie *et al*., 2014a), a process fuelled by the emergence of cheaper and more efficient lighting technologies (Tsao *et al*., 2010; Kyba *et al*., 2014). The large‐scale introduction of such technologies would also be expected to result in changes to the dominant spectral composition of outdoor lighting (Stone *et al*., 2012). However, despite a broad trend of growth in artificial lighting, some locations are becoming darker (Bennie *et al*., 2014a) as lamps are shielded, dimmed, or even removed to reduce light pollution, running costs, and carbon emissions (RCEP, 2009; Falchi *et al*., 2011; Gaston *et al*., 2012). Changes in artificial lighting can impact city performance in a variety of ways (Smith, 2009; Falchi *et al*., 2011), yet many of the potential sustainability impacts remain unexplored (Hölker *et al*., 2010a; Lyytimaki *et al*., 2012). The nature of lighting infrastructure and its operation has obvious implications for energy demands and costs (Gallaway *et al*., 2010; Tsao *et al*., 2010). However, artificial lighting also has numerous positive and negative impacts on social practices and human health; lighting has enabled greater flexibility in the timing of work and leisure activities, although at the cost of disruption to circadian rhythms, behaviours, and physiological processes (e.g. Navara & Nelson, 2007; Falchi *et al*., 2011; Cho *et al*., 2013). Less is known, however, about how natural systems are disturbed and the resulting effects on ecological function and service provision (Rich & Longcore, 2006; Hölker *et al*., 2010b; Gaston *et al*., 2012).

In this study, we focus on ecological impacts of artificial lighting in urban areas and explore how these may vary with different levels of illumination and configurations of the built form.

The value of the semi‐natural components of cities is increasingly recognized, particularly from the perspective of those ecosystem functions with strong links to human well‐being (Carpenter *et al*., 2006; Sadler *et al*., 2010; Haase *et al*., 2014). Given the known effects of artificial lighting on a variety of species and habitats (Longcore & Rich, 2004; Hölker *et al*., 2010b; Gaston *et al*., 2012) and the rapid changes to urban street lighting already underway, research is needed that explores the potential disruption of ecological processes at the city scale. Individuals of most species are sensitive to natural cycles of day and night (Hölker *et al*., 2010b), with light acting both as information and a resource (Gaston *et al*., 2013). For some species, the disruption of these cycles by artificial lighting can impair particular parts of their life history, for example feeding and growth (Boldogh *et al*., 2007), commuting to foraging sites (Stone *et al*., 2009) or the timing of reproduction (Kempenaers *et al*., 2010). Conversely, lighting can bring direct advantages such as concentrating prey (Blake *et al*., 1994; Jung & Kalko, 2010) or for diurnal and crepuscular species, it may extend the hours of activity (Negro *et al*., 2000). A further complication is that lighting may deliver both costs and benefits to a single individual, making the net impact challenging to estimate. For example, artificial lighting has been found to delay roost emergence in the bat *Pipistrellus pygmaeus* (Downs *et al*., 2003), but also to provide foraging locations for the same species (Bartonička *et al*., 2008). Impacts on individual fitness may be sufficient to alter populations and even community composition (Perkin *et al*., 2011; Davies *et al*., 2012), with the potential to affect important ecosystem functions and services such as pollination (Eisenbeis, 2006) or seed dispersal (Lewanzik & Voigt, 2014). However, population or ecosystem‐scale research related to artificial lighting is rare (Gaston *et al*., 2013; Lyytimäki, 2013). One further notable research gap relates to lighting thresholds for ecological impacts and their spatial extent (Gaston *et al*., 2013).

Here, we examine the impact of lighting on animal movement within urban areas as movement is a process relevant to individual fitness, to population resilience and to broader ecosystem structure and function (Nathan *et al*., 2008). Despite the importance of movement for enabling organisms to forage, disperse, and ensure gene flow between populations, the direct measurement of functional connectivity is not always practical (Nathan *et al*., 2008; Zeller *et al*., 2012). Tracking and genetic studies may provide evidence that some patches within a landscape are functionally connected, but on their own, these approaches fail to explain why movement may have been recorded in some contexts but not in others. Understanding the factors that affect movement between habitat patches is therefore important for conservation practice (Rayfield *et al*., 2010; Watts *et al*., 2010), particularly in landscapes undergoing rapid environmental change (Zeller *et al*., 2012). This can be highly complicated as movement may not only depend on patterns of land cover and land use within a landscape, but also on the motivation and ability of individuals to move (Tischendorf & Fahrig, 2000; Nathan *et al*., 2008; Pe'er *et al*., 2011).

The flight behaviour of several bat species may be influenced by artificial lighting (Kuijper *et al*., 2008; Stone *et al*., 2009, 2012; Polak *et al*., 2011) which can cause deviation of the flight path to avoid the most heavily lit area (Kuijper *et al*., 2008; Stone *et al*., 2009), or barrier effects where approaching bats turn and fly in the opposite direction (Stone *et al*., 2009). Barrier effects on commuting bats have also been demonstrated for structures such as motorways that bisect habitat networks (Kerth & Melber, 2009). Several European bat species are known to fly along woodland edges and tree lines when commuting between their roost and feeding locations (Racey & Entwistle, 2003), and the activity of some species is higher with increasing proximity to these corridor features (Verboom & Spoelstra, 1999; Downs & Racey, 2006). This suggests that movement for nocturnal bat species might be simultaneously impacted by the structural fragmentation of habitat networks and by the artificial lighting of commuting routes, both of which are common within urban areas, potentially increasing levels of landscape resistance.

Here, we modelled the effect of both crossing distance and illumination level on the crossing behaviour of a common urban bat (*Pipistrellus pipistrellus*) at gaps in urban tree networks. The resulting model was then used to explore the landscape‐scale implications of different urban lighting scenarios for movement.

The study objectives were to:

1. determine whether the probability of bats crossing gaps in tree lines varies with crossing distance and illumination, and to model any barrier effects;
2. develop spatial models for landscape resistance due to artificial lighting; and
3. explore the implications of these resistance models for habitat accessibility along an urban gradient.

## Materials and methods

**Figure 1:**
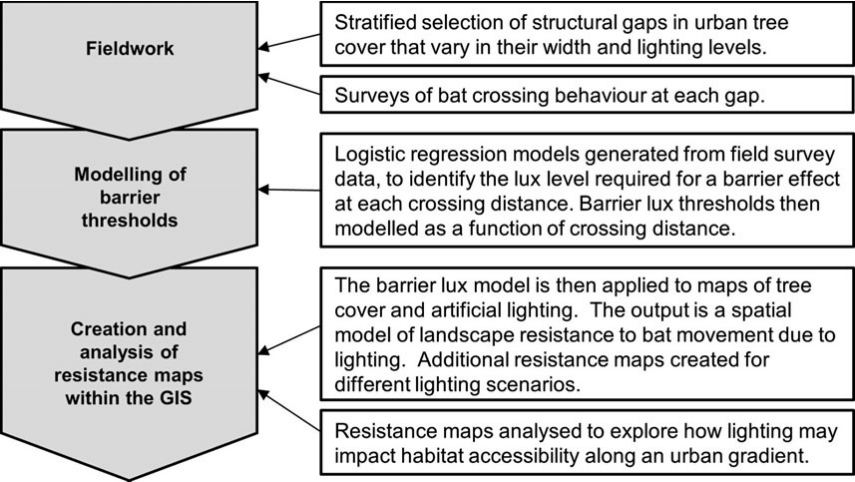
A flow diagram representing key steps within the methods.

These methods are divided into five distinct sections (Fig. 1): (1) the selection of survey gaps within networks of urban tree cover, (2) surveys of gap crossing events by bats, (3) the development of statistical models for gap crossing probability that identify distance‐dependant lighting thresholds for barriers to movement, (4) the translation of this barrier lux model into spatial GIS models for landscape resistance under contrasting lighting scenarios for a case study city, and (5) an analysis of how these scenarios for landscape resistance may impact habitat accessibility along an urban gradient.

### Selection of survey gaps

For *P. pipistrellus*, movement between resource patches is facilitated by linear features such as tree lines (Verboom & Spoelstra, 1999) and therefore, the patch‐matrix‐corridor model (Forman, 1995) would appear to be an appropriate starting point for exploring some of the mechanisms that deliver functional connectivity for this species. A key assumption within this model is that the matrix creates resistance to the movement of individuals between habitat patches and that this resistance is reduced by the presence of linear habitat features that form structural connections between patches. To directly measure functional connectivity between bat roosts and feeding areas within an urban area would be extremely challenging, particularly as both feeding areas and roosts may be difficult to identify or gain access to, given their frequent association with private built infrastructure (Blake *et al*., 1994; Altringham, 2003). This therefore led us to focus on corridor features known to facilitate movement and to explore the degree to which structural gaps in these features and lighting within the intervening matrix could influence crossing behaviour.

Field observations were undertaken in the summer of 2010 within the West Midlands of the United Kingdom (UK), a highly urbanized metropolitan county covering 902 km^2^ with a population of ~2.3 million (S1). *P. pipistrellus* is a species of bat that is broadly distributed over Europe and the Near East (Altringham, 2003), is commonly found within UK cities and can be found throughout the UK West Midlands (Hale *et al*., 2012). It is nocturnal and easily surveyed and was therefore chosen as a model species for exploring the impacts of lighting on bat movement in urban areas. Bats were surveyed at gaps in networks of tree cover, as this species is known to follow the edges of tree lines when commuting between roosts and feeding areas (Downs & Racey, 2006). Tree cover is ubiquitous within the West Midlands, with the exception of the most densely built areas. Trees are typically located along road edges, railway embankments and waterways, in gardens and recreational green spaces, and within the broader amenity planting of commercial areas. Such trees are rarely isolated, but tend to form linear features that follow existing or historic land use boundaries such as the perimeter of a park or residential development. These lines of trees are readily identifiable from aerial photography, and their canopy typically forms a structural network that connects a variety of urban land covers. Despite this high structural connectivity, gaps within this network are evident. Tree lines were selected that were at least 20 m wide and composed of trees >4 m in height, which we consider ideal commuting features for *P. pipistrellus* (c.f. Verboom & Spoelstra, 1999). Gaps in tree lines were defined as locations where a tree line terminated, but where after a break of at least 20 m, a second tree line continued along approximately the same direction. In some cases, it is likely that such tree lines had originally formed a single boundary feature, which was subsequently bisected by the building of a road. Gaps were illuminated to varying levels (S1) by sodium‐vapour street lamps [the dominant source of outdoor artificial lighting within the city (Hale *et al*., 2013)].

**Figure 2:**
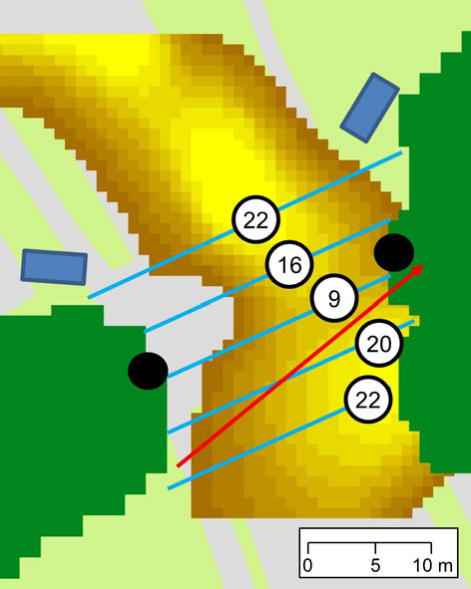
A gap in an urban tree line caused by a road, as represented in the GIS. Dark green areas represent tree cover >4 m high, and the variation in road surface lux is indicated by the yellow gradient. Parallel transects (blue lines) were used to provide an indication of potential crossing routes, the distance of these routes, and the maximum lux that they encounter (indicated by numbered circles). Actual bat crossing routes (red arrow) were mapped based on surveyor (black circle) observations and confirmed based on camera (blue rectangle) recordings.

Our aim was to explore the impact of different gap widths and lighting conditions on crossing behaviour. Rather than experimentally manipulating gap characteristics, we identified a selection of gaps within which to undertake surveys, stratified by width and illumination level. To support this stratification process, gaps were each assigned single values for width and illumination as follows: (1) a variety of gaps in tree lines were identified in arcgis 9.2 (ESRI, Redlands, CA, USA) using a raster layer representing tree cover >4 m in height derived from remotely sensed 1 m resolution colour and near‐infrared photography (2007) (Bluesky International Limited, Coalville, Leicestershire, UK) and LiDAR data (2006) (The GeoInformation Group, Linton, Cambridge, UK). Gaps where the built land cover within a 350 m radius exceeded 60% were excluded, as activity for *P. pipistrellus* tends to be lower in these areas (Hale *et al*., 2012). (2) Measurements of surface illumination within each gap were collected in the field following a 2‐m interval grid of survey points, using a USB2000+RAD spectroradiometer (Ocean Optics, Dunedin, FL, USA). (3) These point measurements were subsequently digitized within the GIS, and spline interpolation was used to generate a 1‐m resolution raster layer representing surface lux within each gap (Fig. 2). (4) Five transect lines crossing each gap were created in the GIS at 5‐m intervals parallel to the main axis of the tree line (Fig. 2), and the length of each transect line was recorded. (5) Each transect line was then intersected with the lux raster to identify the maximum lux encountered, using Hawth's Analysis Tools (Beyer 2004). The result of this process was the calculation of five width and five lux values for each gap (S1). From these, the median width and median lux value were used to characterize each gap, in order to provide typical values to inform the final stratified selection of survey gaps. (6) 27 survey gaps were then chosen to ensure strong coverage across three width and three illumination categories (S1).

### Gap crossing surveys

To record crossing behaviour of *P. pipistrellus,* surveys were undertaken at each gap for a 1.5‐h period following dusk (c.f. Berthinussen & Altringham, 2012). Surveyors were positioned at either end of the gap and used Batbox Duet detectors (Batbox Ltd., Steyning, West Sussex, UK) to be alerted to approaching bats. As directionality of bat detectors is generally poor, it was necessary for surveyors to identify and record the crossing route of each bat, which was later digitized onto the GIS. This species typically commutes at a height of 2.5–10 m (Russ, 1999; Verboom & Spoelstra, 1999; Berthinussen & Altringham, 2012), and individuals were visible when crossing lit gaps. However, when bats crossed in groups or when dark gaps were surveyed, the crossing routes were confirmed using video recordings. Two cameras were used: a Thermovision A20M thermal camera (FLIR Systems, Boston, MA, USA) and a DCR‐HC19E digital video camera (Sony Corporation, Kōnan Minato, Tokyo, Japan) in NightShot mode, with additional near‐infrared (NIR) lighting provided by a 70° angle 850 nm IR LED flood lamp (Camsecure, Bristol, UK). Such lighting is routinely used in bat surveys (Berthinussen & Altringham, 2012), and we found no research to indicate NIR sensitivity for any bat species. The potential for mammals to sense NIR wavelengths has been raised by Newbold & King (2009) and the possibility of NIR lighting impacting bat behaviour should therefore not be excluded, although we emphasize that our research design was systematic across all sites. Bat calls were recorded using a pair of AnaBat SD1 frequency division bat detectors (Titley Scientific, Lawnton, Queensland, Australia) positioned at either end of the gap, allowing each crossing event to be attributed to a species or species group. Calls were identified in analookw (Corben, 2009) using bespoke filters (Hale *et al*., 2012).

### Models for crossing behaviour

**Figure 3:**
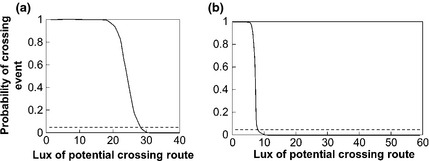
Examples of binary logistic regression models for the probability of gap crossing by *Pipistrellus pipistrellus* at different lux levels. Models are given for crossing distances of (a) 20–40 m and (b) 60–80 m. The dashed lines indicates where the probability of crossing = 0.05.

**Figure 4:**
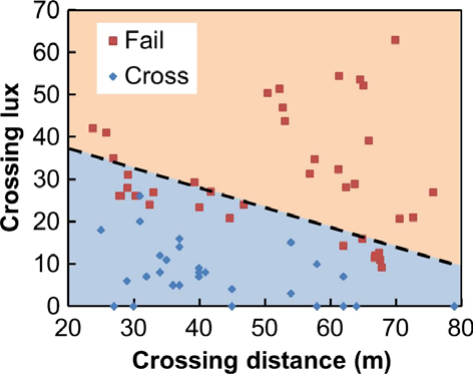
Gap crossing successes and failures for *Pipistrellus pipistrellus*. For crossing events, the distance and maximum lux for each crossing route are plotted (blue diamonds). For gaps where no crossing events were recorded, the distance and maximum lux for gap transects are plotted (red squares). The dashed line represents an estimate of the barrier lux for any given crossing distance, generated using the linear regression equation from the barrier lux model.

Two analyses were undertaken to explore the response of bats to potential crossing routes that differed in their width and illumination level, using data from the gap crossing surveys. The primary analysis sought to identify barrier thresholds for gap crossing, using logistic regression to estimate the probability of a barrier effect (c.f. Awade *et al*., 2012). First, we created a single data set of distance and lux values (referred to as the ‘crossing distance’ and ‘crossing lux’, respectively) for crossing events and failures. For crossing events, these values were extracted from the GIS using the digitized crossing routes. For survey gaps where no crossings were recorded, distance and lux data were extracted from the GIS using the gap transect lines. As lux levels could be highly variable within a gap, we extracted the maximum lux value encountered along each crossing route or gap transect. These data were then used to generate a series of binary logistic regression models in R 2.11.1 (R Core Team, 2014) as follows, using the MASS library (Venables & Ripley, 2002). To explore whether the level of illumination required for a barrier effect (the ‘barrier lux’) varied with crossing distance, subsets of data were selected for modelling using a 20‐m moving window (see Fig. 3 for examples). The barrier lux was defined as the lux level required for a crossing probability of 5% or less. The barrier lux and mid‐range distance from each logistic regression model were then used to model barrier lux as a linear function of crossing distance (henceforth referred to as the ‘barrier lux model’) (Fig. 4).

The second analysis aimed to explore whether the routes taken by bats crossing survey gaps differed from the typical values for the corresponding gaps, in terms of lighting and distance. To highlight potential biases in crossing behaviour, values for the distance of each digitized crossing route were plotted against the median width of the gap being crossed, calculated using distance data from the 5 gap transects. Similarly, the lux of each crossing route was plotted against the median lux value of the gap being crossed.

### Spatial models for the impact of artificial lighting on landscape resistance

Generating resistance surfaces is an increasingly popular way to provide quantitative estimates of how different environmental parameters such as land cover type or human population density may impede animal movement (Zeller *et al*., 2012). Spatial environmental data are typically combined with biological data from surveys to generate cost surfaces that can be interpreted as maps of resistance/barriers to movement. In this case, we created a resistance surface to represent the combined effect of distance from tree cover and illumination by artificial lighting on bat movement. We generated this resistance surface for the city of Birmingham, as it is within the broader West Midlands metropolitan county where our gap surveys were undertaken, and high‐resolution lighting and tree cover data are available for the full extent of the city (Hale *et al*., 2012, 2013). Our aim was to use the barrier lux model to generate a resistance surface using rasters representing distance to tree cover and incident lux as input values for the model variables, from which we could classify the landscape into either accessible or inaccessible patches of land cover. A key assumption within this model was that lighting would have no barrier effect on individuals of *P. pipistrellus* commuting along contiguous tree lines and woodland edges (c.f. Stone *et al*., 2012), but that lighting had the potential to act as a barrier to the crossing of open areas between tree cover.

**Figure 5:**
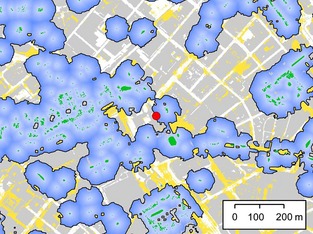
Zones surrounding urban tree cover where the lighting levels are predicted to be insufficient to act as a barrier to movement for *Pipistrellus pipistrellus*. This was derived from a resistance surface generated within the GIS at 1 m resolution, by applying the barrier lux model to a raster representing distance to tree cover (2006/7) and by comparing the output to a map of incident lux (2009). Key: green = trees >4 m, blue network = areas surrounding tree cover where lighting has no barrier effect, blue gradient indicates distance from tree cover, yellow = surface illuminance >20 lx, grey = buildings, and red dot = an urban pond used by bats for foraging. Building outlines derived from OS MasterMap land cover and land use parcels reprinted from original mapping with permission from the Ordnance Survey (2008).

First, the arcgis Cost Distance tool was used to generate a 1‐m resolution raster layer for the entire city representing distance to the nearest tree cover >4 m high. In most cases, the output raster values represented linear distance to tree cover. However, nonlinear distance calculations were also permitted to recognize that euclidian distance measures would be inappropriate for locations where tall buildings would create a barrier to straight line flight at typical commuting height. To achieve this, parts of buildings >30 m in height were selected from the 2008 Ordnance Survey MasterMap (OSMM) land use data set and saved as NoData values within a 1‐m resolution raster layer. All other raster cells were assigned a value of 1, and this layer was then used as an input cost raster as part of the cost distance calculations. Next, the distance value attributed to each pixel was doubled to represent the minimal possible flight distance for a bat leaving and returning to tree cover via that pixel location. This distance layer was then used to calculate the lux level that would be required for a barrier effect at each pixel location, using the Raster Calculator tool to apply the regression equation from the barrier lux model to the distance value of each pixel value. The resulting barrier lux layer was compared to a second layer representing incident lux (2009) for the entire city at 1 m resolution, estimated from aerial night photography (Hale *et al*., 2013). When the lux value for a pixel from the 2009 lighting data set was equal to or greater than the corresponding pixel value within the barrier lux layer, the pixel was classified as inaccessible to our study species. The resulting resistance surface was converted to a polygon layer representing zones surrounding urban tree cover that would be expected to be accessible to bats, based upon the lighting levels in 2009 (Fig. 5). This process was repeated to generate resistance surfaces for two contrasting urban lighting scenarios. The first scenario was for a city without any lighting (the Dark City) and was intended to serve as a baseline model for the independent effect of the structural connectivity of tree cover on landscape resistance. The second was for a heavily lit scenario (the Bright City). This Bright City scenario used the 2009 lighting layer as a starting point, but the surface lighting values of all roads were increased to a minimum of 20 lux, representing a plausible but extreme scenario for future urban road lighting.

### Habitat accessibility along an urban gradient

Urban gradient analyses have been extensively used as a means for exploring the impact of ‘intensification’ on species presence or abundance. Such approaches are a practical response to concerns about the increasing density and extent of urban areas; yet as many ecologically relevant variables covary along such gradients (Hahs & Mcdonnell, 2006), it is rarely clear how these variables combine to drive the ecological patterns observed. The aim of this analysis was to use GIS analyses to explore how the landscape resistance resulting from variations in urban tree cover and lighting could impact habitat accessibility along a gradient of built land cover. Sampling was centred on small ponds (maximum area 2000 m^2^), as these are potential foraging sites for *P. pipistrellus* and are distributed throughout the city. The underlying assumption of this analysis was that ponds would have greater value as foraging habitats if the surrounding landscape had low resistance to bat movement. All ponds within Birmingham were identified from OSMM land use polygons using the GIS and each pond centre was buffered by 350 m, a key spatial scale for predictive models of *P. pipistrellus* activity identified in an earlier study (Hale *et al*., 2012). The percentage built land cover within 350 m of each pond was then estimated using OSMM polygon data, and each pond was assigned to one of seven ‘density classes’ ranging from a low density class of 10–20% built land cover, to a class of ponds surrounded by between 70% and 80% built land cover. Thirty‐five of these ponds were then selected for use in the gradient analysis, 5 from each density class. A greater number of ponds could not be selected without causing uneven sampling, because few ponds were present in heavily built areas.

**Figure 6:**
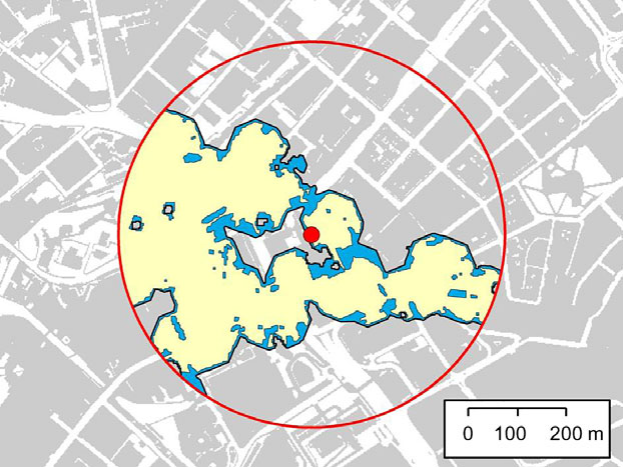
Two spatial models for areas of accessible land cover within 350 m (red circle) of an urban pond (red dot), under a Dark City scenario (blue) and a Bright City scenario (yellow). In this example, when no lighting is present, 44% of the local landscape is predicted to be accessible from the pond, shrinking to 36% in the brightly lit scenario.

The polygon layer representing patches of land cover predicted to be accessible under 2009 lighting levels was then clipped by a 350‐m buffer zone surrounding each pond, and those patches that intersected the pond were retained (Fig. 6). The total area of accessible land cover connected to each pond was then recorded as a percentage of the total surface area within 350 m of the pond. This was modelled against the percentage built land cover within the 350‐m buffer zone using a generalized additive model (GAM) in R 2.11.1, using the MGCV library (Wood, 2006). This process was then repeated for the accessible land cover models generated for the Dark City and Bright City scenarios.

## Results

### Crossing behaviour

**Figure 7:**
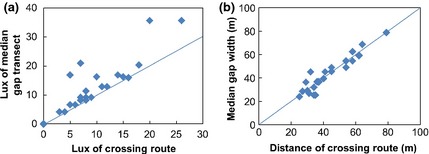
(a) Maximum lux for bat crossing routes vs. maximum lux of the median gap transect. The line indicates where the crossing route lux and gap lux values are equal. (b) Distance of each crossing route vs. the median gap width (based upon gap transects). The line indicates where the crossing route distance and gap width are equal.

The majority of the bats that were recorded crossing gaps were *P. pipistrellus*, and therefore, all results presented here relate to this species. Individuals of *P. pipistrellus* were recorded in the vicinity of all survey gaps, but were only observed crossing 19 of the 27 gaps. The lighting threshold for a barrier effect reduced with increasing crossing distance (Fig. 4), following the linear model: barrier lux = −0.46*crossing distance + 46.2, where the barrier lux is the lux value at which the probability of crossing is 5%. The majority of bats (95.6%) selected crossing routes that were darker than the median gap lux value (Fig. 7a), indicating that bats were choosing to cross in the darker parts of gaps, whereas the length of crossing routes was not consistently larger or smaller than the median gap width (Fig. 7b).

### Landscape connectivity analysis

**Figure 8:**
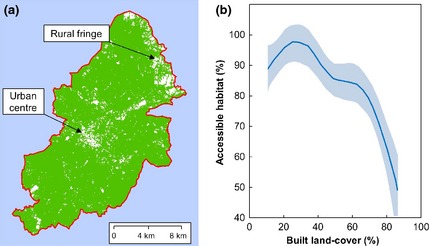
(a) A spatial model of areas within the City of Birmingham where accessibility for *Pipistrellus pipistrellus* is not restricted (indicated by green networks) by artificial lighting levels present in 2009. Accessible land cover is poor in the urban centre and other highly built up areas, as well as at the rural fringe. (b) Estimates of habitat accessibility along a gradient of built surface cover, based on measurements for 35 typical ‘foraging ponds’. Habitat accessibility is defined as the percentage of surface area within a 350 m radius of each pond that the model predicts to be available to bats under a given lighting scenario and that also intersects the pond. Shaded areas represent 95% confidence intervals.

**Figure 9:**
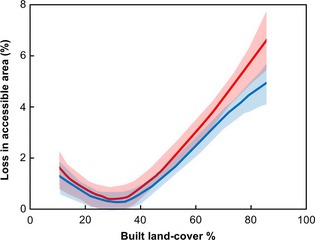
The impact of lighting on the area of accessible land cover connected to urban ponds under Bright (Red) and 2009 (Blue) city lighting scenarios, compared to the levels found under a Dark City scenario.

Landscape resistance for *P. pipistrellus* varied within the City of Birmingham (Fig. 8a) along a gradient of built density (Fig. 8b), as a result of the fine grained arrangement of trees and lighting (S2). When modelled using 2009 lighting data, accessible land cover was highest in areas where built surfaces account for <25% of the landscape, but dropped markedly when built land cover was >65%. Much of this effect is due to the abundance and arrangement of tree cover, although the impact of lighting is clear at higher built densities (Fig. 9). Compared to a Dark City model, lighting levels in 2009 further reduce the percentage of accessible land cover surrounding ponds by up to 5% in heavily built areas, and by up to 7% under a Bright City scenario (Fig. 9).

## Discussion

Outdoor artificial lighting is one of many urban characteristics that are changing rapidly across the globe, yet relatively little is known about its unintended consequences for environmental well‐being. There is a need for research to identify these potential impacts and to contextualize the results in a way that allows mitigation to be targeted effectively. Our analysis demonstrates that lighting can affect landscape resistance in cities, even for a species of bat (*P. pipistrellus*) that that has been recorded in many urban land cover types (Gaisler *et al*., 1998; Hale *et al*., 2012). The greatest impacts on this species are likely to be in brightly lit areas where structural connectivity of tree cover is already low, characteristics typical of heavily built areas such as urban centres.

### Bats, connectivity, and lighting

There is a need to better understand those factors that influence the ability of organisms to move between resource patches and for tools that can predict the impacts of changes at a landscape scale (Adriaensen *et al*., 2003). Central to this is the recognition that functional connectivity of habitats is dependent on both landscape structure and individual behaviour (Tischendorf & Fahrig, 2000). To our knowledge, this is the first study to quantify the effect of lighting on gap crossing in bats and to explore how barrier effects may accumulate across a landscape. Distance thresholds for gap crossing have been identified in the field for groups such as birds (Creegan & Osborne, 2005; Awade & Metzger, 2008) and mammals (van der Ree *et al*., 2004) and then translated into maps of accessible habitat (Awade & Metzger, 2008). However, few attempts have been made to model landscape resistance for bats (but see Frey‐Ehrenbold *et al*., 2013), or to integrate lighting into gap crossing models.

Measures of tree/hedge connectivity have been used to model bat activity in both rural and urban landscapes. A connectivity index for rural trees and hedgerows was developed by Frey‐Ehrenbold *et al*. (2013), and used to identify a positive association between connectivity and activity patterns for three bat guilds. Their results indicate that the distance between patches impacts their likelihood of use. In addition, a connectivity measure used by Hale *et al*. (2012) found a significant effect of connected urban tree cover on bat activity, based upon the assumption that bats could cross gaps in tree cover of <40 m. In both cases, the connectivity model was developed using weightings or distance thresholds chosen to broadly reflect what was known of the species movement ecology, although the results of this study suggests that the inclusion of lighting in such connectivity models could be beneficial.

Researchers have also experimentally tested the effect of lighting on bat movement (e.g. Stone *et al*., 2009) and others have modelled the effect of lighting on the movement of nocturnal species using street lamp locations to create spatially explicit lightscapes (Bennie *et al*., 2014b); however, no studies have considered lighting thresholds for gap crossing. Stone *et al*. (2009) used experimental lighting of rural hedge lines to disrupt movement for the relatively slow flying lesser horseshoe bat (*Rhinolophus hipposideros),* demonstrating a significant barrier effect. In a later study (Stone *et al*., 2012), they found no effect of lighting on *P. pipistrellus* despite using similar illumination ranges to our study. The study by Stone *et al*. (2012) differs to this study in two important ways: firstly in terms of the structural connectivity of the hedges/tree lines (continuous vs. fragmented), and secondly the landscape context (rural vs. urban). It is possible that illuminating a tree line to 50 lux is insufficient to disrupt the commuting behaviour of *P. pipistrellus*, but that the creation of a similarly lit gap may be enough to deter crossing. Moreover, it is possible that a small section of lit hedge in an otherwise dark rural landscape may be of little concern to the fast flying *P. pipistrellus,* whereas the perceived predation risk from crossing a lit gap in an already extensively lit urban area may be high enough to deter crossing.

### Habitat accessibility and urban context

Ecological studies along urbanization gradients are relatively common and typically indicate a reduction in species richness or abundance at high levels of built density (Mckinney, 2008). However, given that many variables such as land cover and disturbance covary (Hahs & Mcdonnell, 2006; Hale *et al*., 2013), it is often unclear which underlying mechanisms are responsible for the ecological patterns observed (Threlfall *et al*., 2011). Here, we found that along a gradient of increasing built land cover, the area of tree canopy cover reduces whilst brightly lit surfaces increase (S2) and that these combine to increase the resistance to movement within heavily built areas.

### Implications for conservation

Relating movement patterns to measures of landscape structure is desirable (Kindlmann & Burel, 2008), particularly as habitat features are often easily mapped. However, it is clear that simple maps of contiguous habitat do not necessarily correspond to functionally connected areas (Tischendorf & Fahrig, 2000) as individuals may move between habitat patches for a wide variety of reasons (Nathan *et al*., 2008), crossing a potentially hostile matrix in the process. Networks of tree cover along with broader elements of ‘green infrastructure’ are commonly recognized in urban planning policy as ‘wildlife corridors’, although the evidence base for their efficacy is mixed (Angold *et al*., 2006; Gilbert‐Norton *et al*., 2010). Whether such structural features actually function to reduce landscape resistance has been a much debated question in landscape ecology (Beier & Noss, 1998). Awareness of the potential impacts of habitat fragmentation (Kerth & Melber, 2009) and lighting (Stone *et al*., 2009) on bat movement has led to a range of mitigation practices, yet in some cases, they appear ineffective (Berthinussen & Altringham, 2012). The ability to commute from roost to feeding areas is crucial to the survival of *P. pipistrellus*, and commuting distances >1 km are not uncommon (Davidson‐Watts & Jones, 2006). It is therefore plausible that restrictions on movement in parts of a city could have fitness impacts at the individual level, as well as limiting the size and extent of urban populations. This highlights the need for a stronger evidence base to support work to protect and improve landscape permeability for urban bats. Whilst bat roosts within the European Union are legally protected under the EU Habitats Directive (1992/43/EEC), the level of protection afforded to commuting routes is less clear (Garland & Markham, 2007). Analyses such as those presented here could support the development of related policy, by clarifying the likely location of commuting routes and the thresholds for their disturbance. These results suggest that networks of urban trees support the movement of *P. pipistrellus*, even when they contain gaps of up to 80 m. However, it is clear that access to feeding habitats may be undermined by lighting within the surrounding landscape, even if the structural elements of the tree network remain unchanged. Although the impacts of lighting demonstrated here are subtle, the approach used to characterize barriers was conservative and lower thresholds for identifying impacts on movement may be more appropriate for conservation purposes. This is supported by the finding that individuals consistently crossed in the darker parts of the gap, even when those gaps were poorly lit, suggesting that all crossing events may be associated with costs (e.g. greater predation risks) that commuting individuals attempt to minimize. The strategic dimming of lights in the vicinity of gaps, combined with the narrowing of gaps through tree planting, might therefore be reasonable conservation measures for this species in urban areas. Such an approach may also have benefits for other bat species that are even less tolerant of lighting such as *Myotis* spp (Stone *et al*., 2012). However, the impacts on *P. pipistrellus* of a broader scale reduction in urban lighting may be more complex, given that this species is able to exploit concentrations of its insect prey surrounding individual lamps (Blake *et al*., 1994). Species of bats may respond to gaps (Kerth & Melber, 2009) and also lighting (Stone *et al*., 2012) very differently; therefore, whilst this approach could be used to model the impact of lighting on landscape resistance for other species, further research is needed to identify appropriate threshold values. Similarly, it is unknown whether the barrier lux model developed here is suitable for all individuals of *P. pipistrellus*, or for different times of the night. Movement is a key component of functional connectivity, and it is important to recognize that a range of factors may influence movement events. Whilst patterns of tree cover and lighting appear to be important, further work is needed to identify how resistance may vary with different land covers or the impact of habitat quality and social structure on movement decisions.

The use of contrasting lighting scenarios to explore potential impacts on landscape resistance could be incorporated into practical conservation measures at a variety of scales. Scenarios are commonly used in sustainability research and practice to test the resilience of infrastructure, communities, resources, and natural systems to a variety of stressors (Nakicenovic & Swart, 2000; Carpenter *et al*., 2006; Hunt *et al*., 2012). The ecological impacts of different scenarios for land cover have been explored by other authors (Adriaensen *et al*., 2003; Kong *et al*., 2010; Sushinsky *et al*., 2013), but we believe this is the first study that has explored the impacts of different urban lighting scenarios at the city scale. This approach may be useful for exploring the impact of specific proposals for changes to urban lighting (Gaston *et al*., 2012) or tree cover (Pincetl, 2010). However, the limited knowledge of how these characteristics can change over time (Gaston *et al*., 2012; Gillespie *et al*., 2012) means that a broader sensitivity analysis may be required to identify network connections that are particularly vulnerable or resilient.

Given the rapid changes underway in cities, urban biodiversity is often faced with multiple ecological disruptors that may be changing simultaneously; disentangling the impacts of these disruptors presents a major challenge. For conservation to shift from a largely reactive to a more proactive approach, it must move on from detecting broad patterns in urban biodiversity to a more mechanistic understanding of the processes that drive them (Mcdonnell & Hahs, 2013). The results of this study indicate that the structural connectivity of tree cover and the levels of lighting within the intervening matrix combine to affect gap crossing behaviour for a common urban bat. In the case study city, this model predicts that as a result, habitat accessibility may reduce with increasing built density, although the potential exists for decoupling this relationship in the future. This has implications for conserving urban biodiversity in cities that are becoming brighter and more densely developed.

## Supporting information


**Figure S1**. Information on survey gap locations and characteristics.

**Figure S2**. Changes in tree cover and lighting along a built density gradient.
