# Supplementary material for: The ecological impact of city lighting scenarios: exploring gap crossing thresholds for urban bats
Source: Glob Chang Biol. 2015 Mar 6;21(7):2467–78. doi: 10.1111/gcb.12884 (PMC4975606; doi:10.1111/gcb.12884)
Supplement: Supplementary file 1 — Figure S1. Information on survey gap locations and characteristics. [file GCB-21-2467-s001.docx]

This Supporting Information file provides additional information on the nature of survey gaps.


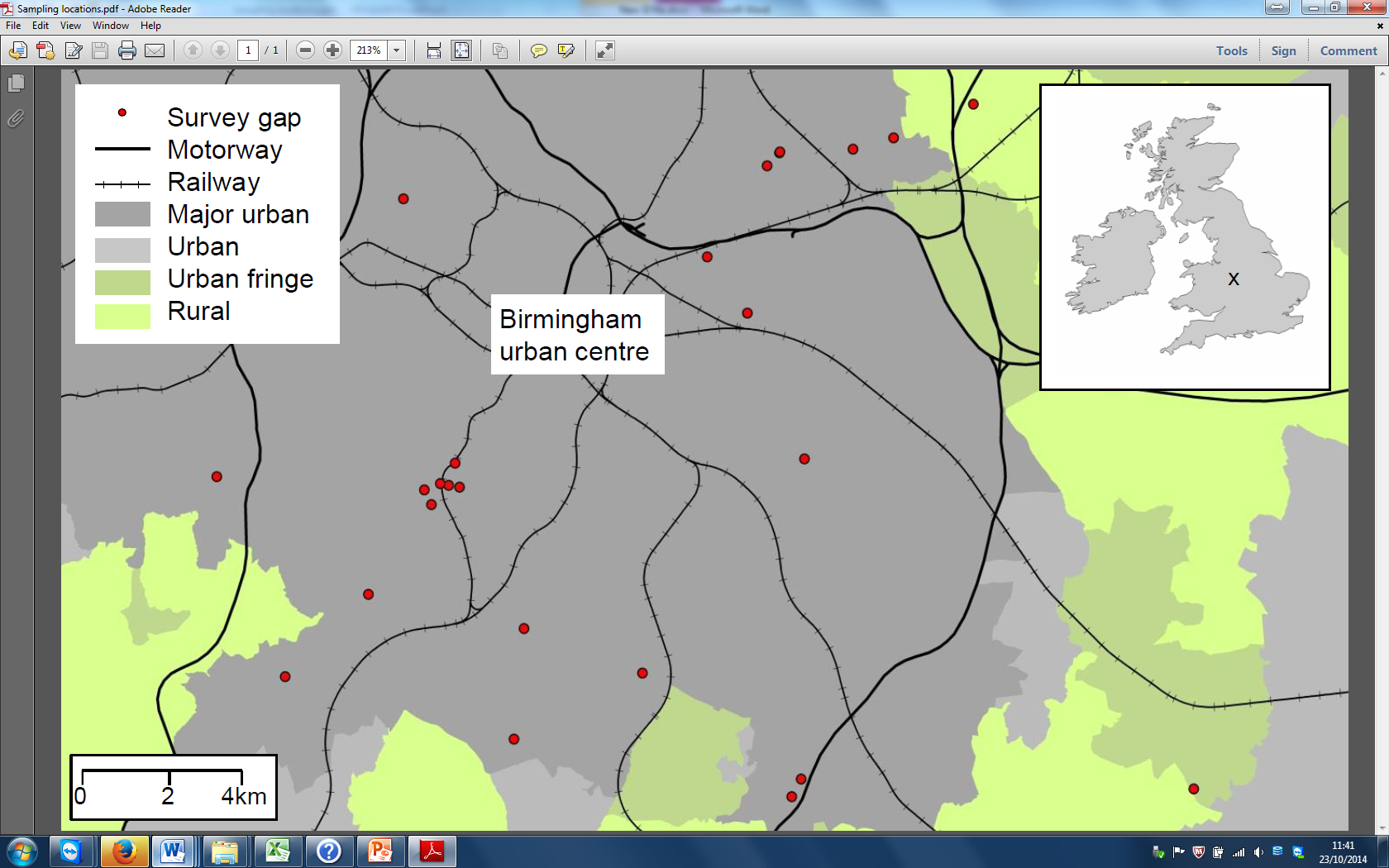


The above figure indicates the position of the 27 survey gaps within the UK West Midlands (inset). The majority of sites are within the City of Birmingham, with some sites selected from adjacent urban areas and the urban fringe. Dense urban areas such as the centre of Birmingham were avoided. The percentage of built land-cover within a 350m radius of each site was <60%.

The above figure illustrates the variety of gap crossing conditions within the survey gaps. Although the 27 survey gaps were stratified by the median gap width and lux, an individual gap could vary considerably in lux (and to a lesser extent width). Lux and distance were measured using 5 transects at each candidate gap, to support the stratified selection of survey gaps. The values for all transect data from the final survey gaps are represented by the blue diamonds in the above graph.

|  |  | **Gap lux** | | |  |
| --- | --- | --- | --- | --- | --- |
|  |  | **Dark** | **0-20 lux** | **20+** | **Total** |
| **Gap width (m)** | **20-40** | 6.5 (0.7) | 4.4(2.2) | 1.3(1.5) | 48 |
|  | **40-60** | 5(0) | 4(1.4) | 0.7(1.2) | 20 |
|  | **60-80** | 6(3) | 0.0 | 0.0 | 18 |
|  | **Total** | 41 | 38 | 7 |  |

Survey gaps were stratified by 9 broad categories based upon their median gap width and lux. This table illustrates the variation in crossing frequency of *P. pipistrellus* within these broad categories. Numbers within the main body of the table represent the average crossing frequency for each gap category, along with the standard deviation in parentheses. Totals for number of crossing events within each width or lux category are given in the final column or row (respectively).
