# Supplementary figures and images for: The ecological impact of city lighting scenarios: exploring gap crossing thresholds for urban bats
Source: Glob Chang Biol. 2015 Mar 6;21(7):2467–78. doi: 10.1111/gcb.12884 (PMC4975606; doi:10.1111/gcb.12884)

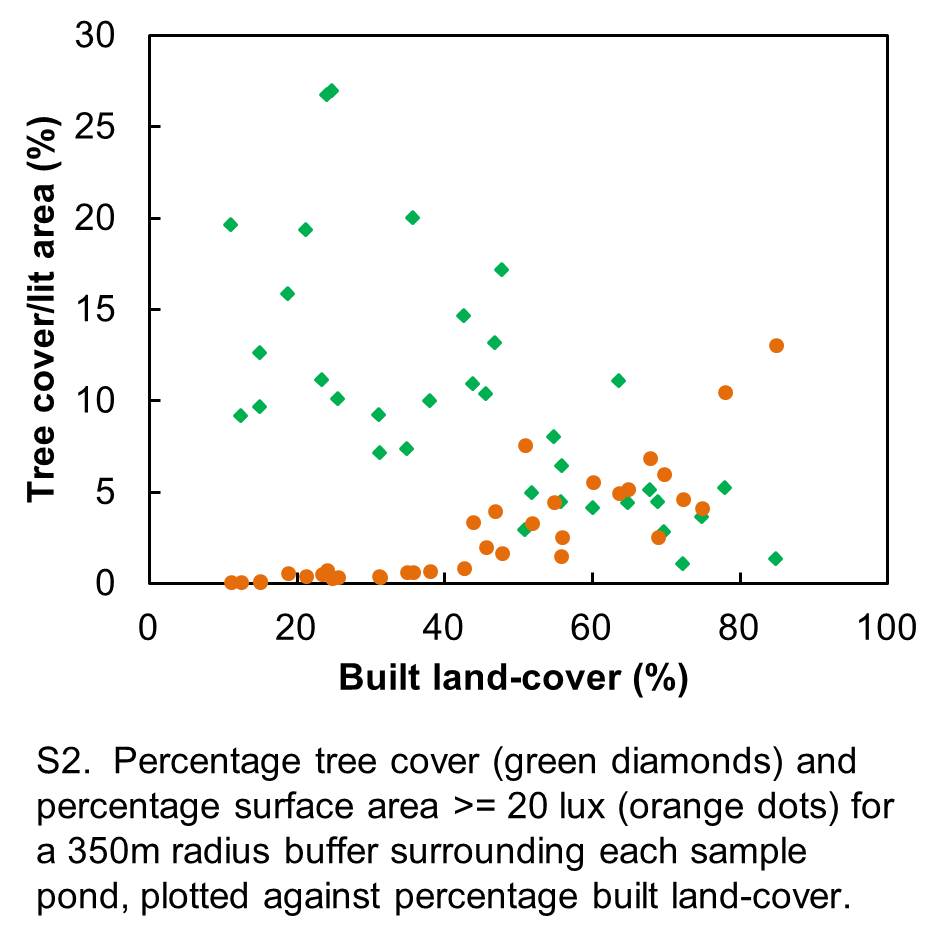

Supplement: Supplementary file 2 — Figure S2. Changes in tree cover and lighting along a built density gradient. [file GCB-21-2467-s002.jpg]
